# Learning as an outcome of involvement in research: what are the implications for practice, reporting and evaluation?

**DOI:** 10.1186/s40900-019-0147-1

**Published:** 2019-03-12

**Authors:** Kristina Staley, Duncan Barron

**Affiliations:** 1 Montague House, 4 St. Mary’s Street, Ross on Wye, HR9 5HT UK; 2 Faculty of Health, Social Care and Education Kingston University, St George’s Campus, 6th Floor Hunter Wing, Cranmer Terrace, London, SW17 0RE UK

**Keywords:** Patient and public involvement, Public involvement, Evaluation, Impact, Good practice

## Abstract

Public involvement in research has evolved over the last two decades in a culture dominated by the principles of evidence-based medicine. It is therefore unsurprising that some researchers have applied the same thinking to involvement, particularly to involvement in research projects. This may explain why they tend to conceptualise involvement as an intervention, seek to evaluate its impact in the same way that treatments are tested, highlight the need for an evidence-base for involvement, and use the language of research to describe its practice and report its outcomes. In this article we explore why this thinking may be unhelpful. We suggest an alternative approach that conceptualises involvement as ‘conversations that support two-way learning’. With this framing, there is no ‘method’ for involvement, but a wide range of approaches that need to be tailored to the context and the needs of the individuals involved. The quality of the interaction between researchers and the public becomes more important than the process. All parties need to be better prepared to offer and receive constructive criticism and to engage in constructive conflict that leads to the best ideas and decisions. The immediate outcomes of involvement in terms of what researchers learn are subjective (specific to the researcher) and unpredictable (because researchers don’t know what they don’t know at the start). This makes it challenging to quantify such outcomes, and to carry out comparisons of different approaches. On this basis, we believe obtaining ‘robust evidence’ of the outcomes of involvement in ways that are consistent with the values of evidence-based medicine, may not be possible or appropriate. We argue that researchers’ subjective accounts of what they learnt through involvement represent an equally valid way of knowing whether involvement has made a difference. Different approaches to evaluating and reporting involvement need to be adopted, which describe the details of what was said and learnt by whom (short term outcomes), what changes were made as a result (medium term outcomes), and the long-term, wider impacts on the research culture and agenda. Sharing researchers’ personal accounts may support wider learning about how involvement works, for whom and when.

## Introduction

Public involvement^1^ in research has evolved over the last two decades in a research culture heavily dominated by the principles of evidence-based medicine (EBM). EBM recommends combining clinical experience and patient values with the best available research evidence to inform decisions about an individual’s care [1]. It is therefore unsurprising that researchers who work in this culture have often sought to understand and implement public involvement through the same lens. This may explain a tendency for some researchers to think about involvement as an intervention, to seek to evaluate its impact in the same way that treatments are tested, to highlight the need for an evidence-base for involvement, and to use the language of research to describe its practice and report its outcomes.

In this article, we explore how this thinking may be unhelpful and can contribute to misunderstanding and poor practice [2]. We suggest an alternative conceptualisation of involvement: as a conversation that supports two-way learning. We draw on our own experiential knowledge as people who have provided practical support to researchers and the public, developed policy and guidance and evaluated involvement. Our approach is rooted in the expertise and experiences of the many people we have worked with over the years, rather than being theory or evidence-based. This is not a report of research, but a commentary that offers an opinion for further debate.

We first consider what researchers and the public learn from involvement and how the short-term outcome (learning from conversations, which is subjective and difficult to quantify) leads to the commonly described medium-term outcomes (objective changes to research design, delivery and dissemination) and the impacts of involvement (long-term and broader changes to the research culture and agenda) [3, 4]. We particularly focus on the key features of researchers’ learning, as we believe this is what most challenges current thinking about involvement.

## Plain English summary

Public involvement has evolved in a research culture that places great emphasis on using the best quality research evidence to inform decisions about healthcare. It is therefore unsurprising that researchers have tended to apply the same thinking to involvement. This may be why some researchers state that involvement needs to be evaluated using the same methods that are used for testing treatments and reported in the same way as research findings. We explore why this thinking may be unhelpful. We suggest involvement is better understood as ‘conversations that support two-way learning’. When described this way, there is no particular ‘method’ of involvement but a range of approaches that need to be tailored to the situation and the needs of the people involved. The quality of the interactions becomes more important than the process. All parties need to be better prepared to learn from each other. The immediate outcomes in terms of what researchers learn are subjective (specific to the researcher) and unpredictable (because researchers don’t know what they don’t know at the start). We believe this may make it impossible to develop an evidence-base for involvement in the same way as for treatments, and that researchers’ accounts of impact are as good a way of knowing whether involvement has made a difference. Sharing these accounts may support wider learning about how involvement works, for whom and when.

### Involvement as a conversation that supports learning

Patients and carers gain experiential knowledge through their direct experience of living with, or caring for someone with a health condition and/or using health and social care services [5]. This is knowledge that people *without* that particular experience will lack, including researchers [6]. Researchers acquire text-book knowledge of the condition they work on [6] and their area of research expertise, e.g. statistics, as well as experiential knowledge of conducting research. (If the researchers are also clinicians, they will have text-book and experiential knowledge of delivering services and care.) Involvement in research is in essence a conversation between these individuals in which they share their expertise and experience, particularly when researchers and the public work together on research projects. The immediate outcome from this dialogue is learning, defined as gaining new knowledge, skills and values which leads to different choices, actions, behaviour [7]. Through mutual learning [8], researchers and the public ideally reach joint decisions about what (or what not) to research and how best to do it, with the ultimate, shared goal of generating high quality evidence to improve services and care.

### How do the public benefit from this mutual learning?

Typically, the public report gaining the following from involvement [9–12]:

- knowledge about how research works
- knowledge about the latest and best evidence relating to treatment and care
- confidence and new skills (e.g. communication skills, presentation skills, influencing and persuading skills)
- new ways of coping and managing their health condition

This learning has multiple benefits. An immediate outcome is that the public gain a better understanding of the research context, which helps them to make more useful contributions during conversations with researchers [6, 13]. For example, on learning that all the items in a quality of life questionnaire needed to be answered to make the measurement valid, a patient understood what was missing from the instructions for the participants, and was then able to improve the wording [6, 14]. Wider impacts for the individuals involved include gaining confidence to return to work after a period of ill-health [10, 11], and being able to apply their new knowledge in other contexts, for example in developing health and social care services and policy [15].

### How do researchers benefit from this mutual learning?

Typically, learning from others’ experiential knowledge helps researchers to [3, 4, 14]:

- develop new ideas for research
- choose between alternative directions for research
- identify otherwise unanticipated problems, as well as solutions to overcome them
- confirm the right decisions have been made, thus instilling confidence in those decisions
- understand what matters most to patients/ carers and the public, providing a rationale for a project as well as personal motivation

If researchers make decisions about their research *without* involvement, it is easy for them to make assumptions or miss significant issues [3, 16]. Involvement fills the gaps in researchers’ knowledge and corrects assumptions, thus avoiding bias in their thinking. This can happen at any stage in the research cycle [17], because a researcher’s lack of experiential knowledge has the potential to influence *any* and *every* decision they make. This is why the conversation with the public needs to be ongoing, and not restricted to one or a few stages of research [18].

### Key characteristics of researchers’ learning

Researchers’ learning has two key characteristics. Firstly, it is subjective, and secondly it is unpredictable, as illustrated by the following example:

“*A new research proposal on carpal tunnel syndrome was presented to the RUG* [Research User Group] *and a discussion ensued about the key questions that the research should address. A RUG member explained that she lost her job… because the condition stopped her from doing fine finger movements. The researchers realised that they had not considered the importance of remaining in work and being economically active within their proposal. Thus, the one story raised awareness of a major area of investigation that was therefore included in the (successful) bid*”. (Reference [19], page 151).

In summary, an immediate outcome of the conversation with the RUG was that the researchers learnt about how work life is affected by carpal tunnel syndrome. The medium-term outcome was that they changed their proposal, which may have contributed to securing funding. The wider impact was that the research project better reflected the reality of patients’ lives [4].

An initial response to this example may be surprise that the researchers had not considered how a health condition can affect employment, when this is a common experience. Perhaps these were junior researchers who needed to learn this for the first time. Or perhaps they were experienced researchers who had wrongly assumed that carpal tunnel syndrome would not cause someone to lose their job. What the researchers were initially thinking is unclear and would be valuable information to include in future reports. However, it is clear that these researchers learnt useful information that influenced *their* specific thinking and plans.

This example illustrates how each researcher may be in a very different place in terms of what they need to learn from involvement. It depends on what the individual ‘doesn’t know’ at the start. This is true for all researchers, irrespective of their level of experience. A professor lacking experiential knowledge is just as likely to make assumptions as a PhD student [6, 20]. If the researchers in the example above had already considered work-life in their proposal, then the involvement wouldn’t have led to the same learning outcome. What any one researcher is likely to learn from involvement will evolve over time, both within a single study and from project to project.

Researchers often ‘don’t know’ what they ‘don’t know’ [21]. They may approach involvement with some ‘known unknowns’, for example, questions such as ‘Is my questionnaire an acceptable length?’, or ‘Is my patient information leaflet easy to understand?’, while being unaware of any assumptions they are making elsewhere. These won’t come to light until they speak with the public. This makes the outcome of involvement unpredictable. While it is generally known how involvement can make a difference, it is not possible to predict what it will achieve for any specific researcher or project [21]. What one researcher learns from involvement, may not be useful to a different researcher working on a different project, so the ‘findings’ may not be generalisable ‘data’ [22, 23], but highly personal and context-specific learning.

### Implications for involvement practice

With current conceptualisations of involvement, standardising practice seems to be important to some researchers to ensure involvement is consistent and effective. Researchers also want to compare approaches and often ask ‘What method should I use for involvement?’ When the public are perceived as *the intervention*, much attention is given to getting them ‘involvement-ready’. In the remainder of this section, we explore the implications of this thinking, and the alternative approaches suggested by reframing involvement as conversations that support learning.

### Is there a ‘method’ for involvement?

A high-quality research method is controlled so that the researcher decides where the research will take place, at what time, with which participants and in what circumstances. It usually follows standard procedures, which are systematic (follow a fixed plan), replicable, verifiable and often empirical (measured and quantified). We conclude that few or none of these attributes of a research method are applicable or relevant to involvement in research.

By way of contrast, a researcher cannot control involvement because the process is constantly changing, and needs to be flexible and responsive to the context. For example, the way that researchers need to talk with residents of care homes may be very different to the way they need to talk to teenage boys with attention deficit disorder. The way they need to talk to people if they simply want to explore some new ideas (e.g. talking over a cup of coffee), may be very different to the way they need to talk to people when they need to make a formal decision with multi-stakeholder agreement (e.g. making a decision about which inclusion and exclusion criteria to use in a clinical trial).

Similarly, the immediate outcomes of involvement cannot be replicated or verified, because if two different researchers carried out the same ‘method’ of interacting with the same public, their learning could be entirely different. Nor can involvement aim to be systematic, because what researchers learn initially might change their thinking and direction. Involvement is evolutionary, in unpredictably progressing through a series of interrelated episodes of learning, rather than following a linear, fixed path.

We conclude there is no ‘method’ for involvement, but a range of techniques which may be more or less appropriate in different contexts, and importantly need to be negotiated and agreed on every occasion with the specific individuals involved.

Our experience suggests that researchers have sometimes assumed that there is such a ‘method’. The current trend seems to be to ‘set up a group’ and then meet with them three times, once at the beginning to get input into the design, once at the middle to discuss any ongoing problems and once at the end to interpret the findings and agree how to disseminate the results. We note that this is only one possible approach and there are many others which may be more appropriate for different contexts [24]. The answer is not always a committee.

While standardising the approach may not be necessary, we do want to emphasise that we fully support the development of national standards for involvement in the UK [25] as well as ethical approaches [22] to involvement. By aiming to define what good involvement looks like, these standards ensure the quality of the process, without specifying what form that process should take. With our focus on learning, the standards help to create the conditions in which successful learning conversations can take place. They help researchers and the public to be clearer about the nature of the conversation (by clarifying roles and expectations). They help create the conditions that encourage open and honest dialogue i.e. valuing the public’s contributions [8], developing mutual trust and respect [26], being ethically conscious [22], creating safe spaces for sharing potentially shameful or painful experiences [27] and preparing people for what will happen through training and support [8]. We conclude that following best practice in involvement often means ensuring the quality of the interactions between researchers and the public, rather than being precise about the ‘method’.

We suggest involvement is more akin to the commonplace, ongoing dialogue between researchers, using a variety of processes that are responsive to immediate information needs. No researcher asks ‘What method should I use to talk to my colleague?’ We believe making the links between involvement and what researchers already do to collaborate with others has the potential to help researchers understand *how* to do involvement.

### Are the public the ‘intervention’?

Conceptualising the public as the intervention can lead to a desire to standardise their contributions to ensure quality, in the same way that the quality of data is assured through appropriate sampling. Researchers can assume that a representative group must be involved that somehow reflects broad demographics such as gender, age, ethnicity and sometimes geography. However, by way of contrast, when involvement is conceptualised as learning conversations, then the key issue becomes ensuring researchers can tap into the relevant experiential knowledge to learn what they need to learn. This doesn’t always relate to representativeness, but to having specific experience(s).

This raises the question ‘Who has the most relevant experiential knowledge in any given context?’ This is not easy to answer, because at the start, researchers ‘don’t know what they don’t know’, and therefore cannot be certain who possesses the insights they need. We suggest the solution is to involve people with *diverse experiences* of the topic under investigation, as this will include people with a range of potentially relevant knowledge. Starting the conversation with the public may help researchers check their assumptions about whose experience is relevant in their context. Sometimes it may be about involving people with diverse backgrounds, but sometimes this could be about talking to people who are housebound rather than able to travel [6], or people who work or are retired (as above). The nature of the research question and the decision being made will determine who needs to be involved. We believe the issue merits further investigation, to help researchers answer this question for themselves.

### Gaps in current guidance and improving practice

Much of the current guidance and policy for involvement in research places great emphasis on the public - recruiting, training and supporting them [17]. Much less attention is given to preparing researchers, in terms of the soft-skills they may need for involvement. However, when involvement is understood as a conversation that supports two-way learning, researchers’ effective participation is 50% of the equation. The lack of attention to researchers’ training then seems to be a major omission. We believe better preparing researchers for involvement may be highly significant to improving its practice [8].

On occasion we have observed examples where a public involvement group has been established and then left alone to be managed by a member of staff, without any plans for conversations with researchers (unpublished observations). Some of the researchers involved assumed this would be ‘gold standard’, as it would constitute being ‘patient-led’ in line with previous descriptions of the level of involvement [17]. However, when involvement is understood as a process of two-way learning, dialogue with researchers becomes fundamental to the whole process. Furthermore, since each individual researcher may learn something different from conversations with the public, it can be argued that all the researchers in a team need to be part of the ongoing dialogue. If the responsibility for involvement is delegated to a single researcher or staff member, this could limit its potential for impact [8].

Taking part in and learning from conversations requires certain skills and contributions from all parties. Everyone needs to be prepared to listen and learn, to share values and experiences and to change their own thinking and behaviour. Everyone needs to expect constructive conflict that will support the development of new ideas. Therefore, the public may need to learn to become effective critical friends. Researchers may need to learn how to become ‘listening researchers’ [28], being open to criticism, avoiding defensiveness and being willing to respond to the public’s input. However, researchers also need to be able to critique the public’s ideas, which might need revision or may even be rejected, if their implementation would negate the fidelity of the research [11]. Developing relationships based on mutual respect and trust [8, 26] are important in creating an environment in which criticism can be viewed constructively, and offered and received without hostility.

We note that involvement leads, the staff who support involvement, often play an essential role in facilitating effective dialogue between researchers and the public. They provide a ‘translation’ service, challenge stereotypes, act as an independent power broker, as well as managing and supporting everyone involved and the process [28, 29]. As recommended by the NIHR’s review of involvement in 2015 [30], we believe this role merits further evaluation to understand: a) how these staff can best support two-way learning, and b) to inform their personal and professional development.

### Implications for evaluation

Current conceptualisations of ‘the public as the intervention’ suggest that the question that needs to be answered through evaluation is along the lines of ‘Do the public make a difference?’ When involvement is understood as two-way learning, this question no longer makes sense. The key question is then ‘Does the interaction between researchers and the public lead to a change?’

Some researchers also conclude that involvement needs to be evaluated in the same way as other interventions, for example through randomised controlled trials (RCTs), in order to collect quantifiable, objective evidence of impact and support the development of an evidence-base. In this section we discuss how these goals may be problematical.

### Is an evidence-base for involvement necessary?

Many reports of involvement conclude that the evidence published to date is insufficient and ‘anecdotal’ [27]. This is consistent with the norms of testing and developing clinical interventions. However, ‘evidence’ has a particular meaning in the culture of EBM, which is diametrically opposed to experiential knowledge as described in Table 1.

**Table 1:** Evidence versus experiential knowledge in the context of EBM

The nature of ‘evidence’	The nature of experiential knowledge
Data obtained through systematic enquiry	Knowledge gained through experience – wisdom
Objective	Subjective
Rational	Emotional
Written in scientific and technical language	Written in the individual’s own words
Quantitative or qualitative data	Descriptive, personal accounts

In the field of public involvement, it is understood that there are different ‘ways of knowing’. Researchers who dismiss the public’s contributions as anecdotal are encouraged to see the value of the wisdom in those people’s experiences. When involvement is conceptualised as a learning conversation, then mirror processes become apparent. Researchers are exposed to the public’s experiential knowledge through involvement and take away their own subjective learning from the experience. Researchers’ reports of the outcomes and impacts are therefore personal accounts of the insights they have gained, and descriptions of what changed as a result. It is inconsistent to criticise these researchers’ reports as ‘poor evidence’ when the public’s personal accounts are recognised to have value. Researchers’ ‘stories’ therefore represent another way of knowing whether involvement has made a difference.

### Does the outcome of involvement need to be measured?

Researchers often state the need for outcomes of involvement that can be quantified [31]. Again this is the norm and entirely appropriate within the field of EBM. Measurement is important in this context because it allows researchers to:

- Assess the statistical significance of a precisely defined outcome e.g. whether a particular % reduction in blood sugar is clinically meaningful
- Predict the likelihood of an outcome e.g. to be able to say the outcome is achieved in 60% of patients who receive this intervention
- Make comparisons across interventions by using the same precisely defined outcomes in different trials

It remains unclear whether these are relevant and meaningful goals for involvement.

The first challenge is that it is difficult to quantify learning from experience. It is possible to quantify the learning of text-book knowledge, through exams that test whether certain facts have been assimilated. However, with experiential learning, there is no fixed set of facts to learn. In the carpal tunnel syndrome study above, how could the researchers’ learning about the impact of a health condition on work-life be quantified? It can only be described.

In some contexts it may be possible to measure the medium-term outcomes that result from what researchers learn, for example, an improvement in recruitment to research [10, 11]. Developing such impact measures may be possible, but may not be useful in predicting outcomes [21]. Knowing that involvement has had a measurable impact on recruitment in one project, may have little bearing on whether involvement will produce the same outcome in a different study, with different researchers.

Similarly, making comparisons across different approaches to involvement are meaningless, when one of the key factors influencing impact – the starting point of the researcher – is not constant. Even if different approaches to involvement were tested with the same researcher, the outcome would change each time. Using the example above, the researchers only needed to learn once about the impact of a health condition on work. If they learnt this fact through one approach to involvement, it would be impossible to test whether any other approaches were better or worse in terms of achieving this outcome.

These challenges may explain why, despite much interest in the issue for many years, impact measures for involvement have not been developed [32]. Nor is it clear whether such a form of evidence would in fact add value to the field. Fuller and more in-depth accounts of researchers’ learning (see below) may prove more effective in increasing our understanding in ways that could usefully inform involvement practice and policy, and address questions about how the process works.

### The limitations of using (RCTs) to assess impact

RCTs have been used in a few instances to assess the impact of involvement. The results have shown either no statistical difference or a very tiny statistical difference [33, 34]. The inherent problems with using an RCT in this context can be explained by understanding involvement as a learning conversation.

RCTs are based on PICOT formatted research questions, where there is a defined population (P), intervention (I), comparator (C), measurable outcome (O) and fixed time (T) for collecting data. The question that previous RCTs have addressed is ‘Does public involvement in research lead to better recruitment when compared to research projects without involvement?’ [33, 34]. These studies have made comparisons within the portfolio of a research organisation (e.g. all the studies supported by a specific research network) and compared different aspects of recruitment between the studies either with or without involvement. They included a measurable outcome (recruitment) and a comparator (involvement versus no involvement). However, the nature of the intervention was not considered. All approaches were grouped together, as if there is only one way to do involvement, when in fact it can take many different forms depending on what precisely researchers ask the public to do [17].

Furthermore there was no attempt to define the population in these studies, which would be ‘projects in need of help with recruitment’. Including all the research projects within a portfolio in such a comparison is like including all patients with any health condition in a clinical trial testing a drug for arthritis. There will have been projects included in the comparison where recruitment was not an issue. This helps to explain why the statistical analysis in these RCTs revealed a barely significant impact on recruitment [33, 34], in contrast to researchers’ personal accounts which often describe dramatic improvements [35]. We conclude that it is difficult to identify which research projects have a problem that involvement could solve *ahead of time*, as the issues are often ‘unknown unknowns’ for the researchers. This makes using RCTs to assess impact problematic.

By focusing on the short term outcomes for *research*, such as improved recruitment, there’s a risk of missing the perhaps more significant, longer-term impacts on the wider research agenda and culture. One of the main purposes of involvement is to make research more relevant to the end-user and therefore finding ways to assess whether this is genuinely happening seems to be important, even if this takes more time.

The question remains whether RCTs have added any new understanding of involvement beyond what researchers’ accounts have previously described. Have they ‘proved’ involvement produces this outcome? Have they explained ‘how’ involvement impacts on recruitment? Do they help predict ‘when’ involvement will help with recruitment? Such questions may be better answered through a qualitative analysis of the many published accounts of researchers’ experiences of where involvement improved recruitment. Identifying the contextual factors that were significant, especially the gaps in the knowledge and assumptions made by researchers, could help explain *when* involvement helps with recruitment and how this is most often achieved. Furthermore, there is a risk that research funds are wasted on trying to evidence what is common sense. For example, is an RCT necessary to show that rewriting technical information in plain English results in text that is easier for a non-technical audience to understand? [36].

### Reporting the impact of involvement

Reporting the impact of involvement has been described as weak and in need of improvement to inform practice and aid understanding of how it works [10, 11]. However, attempts to date to improve reporting, e.g. the GRIPP2 checklist, have drawn on guidelines relevant to reporting research methods and findings [37]. Research reporting typically aims to be independent of the individual researcher who carried out the research, so that the findings are objective and generalisable. If the same approach is applied to reports of involvement, then the learning experiences of the researchers are missed out. Only the objective, observable changes to research are described. We believe this limits understanding of *how* the involvement made a difference, and fails to explain why the outcome of involvement for any particular individual or project cannot be certain.

There are no precedents or existing guidelines to draw on to describe the individual researcher’s experience of conducting research. The researcher’s journey of ideas, the twists and turns in their thinking, the experience of reaching dead-ends, solving problems and all the conversations along the way are not typically part of a final report. Researchers have many collaborators who influence all kinds of decisions during a research project, but their input is rarely described. We therefore conclude that new approaches to reporting involvement need to be developed that enable researchers to ‘tell the story of what happened’. These need to describe where the researchers started, what they learnt from their conversations with the public, what changed as a result, both immediately in terms of objective practical changes to their research, and more long-term, wider impacts on all the people involved and the research culture and agenda [4].

## Conclusion

This article has explored the challenges that arise if involvement is conceptualised and evaluated in the same way as an intervention, and reported in the same way as research findings. We suggest that public involvement is better understood as a conversation that supports mutual learning between researchers and the public, and that this leads to different conclusions (summarised in Table 2). This may be a challenge for the dominant research culture which often values objectivity over subjectivity and evidence over experience.

**Table 2:** Key messages from conceptualising involvement as a conversation that supports learning

• Involvement in research is essentially a conversation where researchers and the public share their knowledge, values and opinions to learn from each other	
• Researchers’ learning from others’ experiential knowledge is subjective and unpredictable, making measurement of this outcome difficult	
• The conversation needs to be ongoing, formal and informal, to avoid bias in researchers’ thinking in every decision they make about their research	
• The approach doesn’t need to be standardised – it doesn’t always mean setting up a group in the same way every time	
• The quality of the interaction between researchers and the public may be more important to support learning than the precise process	
• Objective reporting of the outcomes and impact of involvement do not provide the full picture because researchers’ learning is missed out	
• New approaches to evaluation and reporting impact of involvement may be required to include researchers’ personal accounts of their experience and the wider impacts on the research agenda and culture	

We further conclude that current conceptualisations of involvement may be causing confusion for some researchers and may even lead to poor practice. It seems researchers are still left unclear about the purpose of involvement in their own work and how precisely to do it [38]. Involvement seems to have become overly complicated and mysterious, with the only solution being touted as ‘better evidence’. Our view is that involvement is in fact an ordinary and everyday experience of researchers, who are constantly learning from a wide range of collaborators. Such conversations help to improve ideas, help researchers to make better choices and decisions, and help to solve their problems. This is part of the individual’s subjective experience of conducting research. Therefore trying to standardise involvement processes as ‘methods’ and to objectify the outcomes, may be akin to ‘forcing a square peg into a round hole’. The richness and value of subjective learning needs greater recognition.

A crucial question to ask about the concept of learning from other’s experiential knowledge is ‘How is working with the public different to working with other types of collaborator?’ We believe the challenges lie in working with people who are not part of the research system, whose knowledge is not standard or text-book, who don’t speak the same language, and may not work according to the rules and norms of research organisations. Improving involvement policy and practice might therefore require researchers to change their expectations and assumptions. They may benefit from finding ways to engage in the creative conflict that supports learning, to learn to work in ways that differ from their usual practice and with very different kinds of people, and to accept that the process will not be methodical, and the outcome will be unpredictable. Researchers may need to learn to trust their subjective experiences of how involvement helped them as individuals and to recognise this represents an equally valid way of knowing that involvement has made a difference. Sharing their personal accounts may support wider learning about how involvement works, for whom and when. This could help to improve the opportunities for learning for everyone involved and better prepare all parties to work together as a creative- thinking team.

## Abbreviations

EBM: Evidence based medicine
PhD: Doctor of Philosophy
RCT: Randomised controlled trial

## Footnotes

1 We use the term public to refer to people with relevant experiential knowledge (knowledge gained through experience) to contribute to research. Depending on the context, this could be patients, carers, potential and current users of services and/ or community members. We use the term involvement to refer to the activities whereby these people act as collaborators in research influencing its design, delivery and dissemination.
